# Digital transformation and emerging technologies in healthcare and pharmaceutical supply chains: a bibliometric mapping review

**DOI:** 10.3389/fdgth.2026.1884773

**Published:** 2026-08-27

**Authors:** Gulden Zhalbirova, Zhalgaskali Arystanov, Kateryna Zupanets, Zaru Kapassova, Madina Syzdykova, Kymbat Bekmuratova

**Affiliations:** 1 Department of Pharmaceutical Disciplines, Astana Medical University, Astana, Kazakhstan; 2 Department of Pharmaceutical Medicine, MEDICE Arzneimittel Pütter GmbH & Co. KG, Iserlohn, Germany; 3 Department of Children Diseases №1, Astana Medical University, Astana, Kazakhstan

**Keywords:** artificial intelligence, bibliometric analysis, blockchain, cold chain, digital transformation, healthcare supply chain, internet of things, pharmaceutical logistics

## Abstract

**Background:**

Scientific output on digital transformation in healthcare and pharmaceutical supply chains increased substantially after 2020, indicating growing research attention to resilient and digitally integrated logistics systems. However, the literature remains fragmented across technologies such as blockchain, artificial intelligence, Internet of Things, predictive analytics, cold chain monitoring and healthcare logistics optimization.

**Methods:**

This study conducted a bibliometric analysis of scientific publications related to digital transformation and emerging technologies in healthcare and pharmaceutical supply chains. Data were retrieved from Scopus, PubMed and Web of Science databases following PRISMA 2020 screening principles. After duplicate removal and eligibility assessment, 83 peer-reviewed English-language journal articles published between 2015 and 2026 were included in the final analysis. Bibliometric mapping and thematic analysis were performed using VOSviewer and Bibliometrix/Biblioshiny.

**Results:**

Within the analyzed corpus, the results showed a substantial increase in scientific publications after 2020, consistent with growing research attention to resilient and digitally integrated healthcare supply chains. Blockchain showed the highest visibility in keyword and citation-based analyses, particularly in relation to traceability, transparency and anti-counterfeit systems. Additional major research areas included artificial intelligence, predictive analytics, IoT-based cold chain monitoring and healthcare logistics optimization. Thematic analysis identified strong literature-based associations between digital technologies, supply chain resilience and pharmaceutical traceability systems.

**Conclusions:**

Digital technologies are increasingly represented in research on healthcare and pharmaceutical supply chain transformation. The findings suggest that blockchain, AI and IoT technologies may support transparency, traceability and operational resilience. However, implementation barriers related to interoperability, infrastructure costs, data privacy and regulatory complexity remain significant challenges. These findings should be interpreted as bibliometric and thematic patterns within the analyzed English-language journal literature rather than direct evidence of technology implementation effectiveness.

## Introduction

Healthcare and pharmaceutical supply chains have become increasingly complex because of globalization, strict regulatory requirements, counterfeit medicine risks, logistics disruptions and the need to maintain cold chain integrity. In recent years, digital technologies have been increasingly discussed as tools that may support transparency, traceability and operational efficiency across healthcare logistics systems. The COVID-19 pandemic further exposed vulnerabilities in healthcare supply chains and increased research attention to resilient, data-driven and digitally integrated logistics infrastructures (1–3). Blockchain technology has received substantial attention for traceability and anti-counterfeit applications. At the same time, artificial intelligence, machine learning, big data analytics and Internet of Things technologies are increasingly explored for forecasting, inventory optimization, real-time monitoring and decision support (4–6).

In pharmaceutical logistics, digital transformation is particularly important because temperature-sensitive medicines and vaccines require continuous monitoring during transportation and storage. IoT-based sensors and monitoring platforms are discussed in the literature as tools that can support real-time temperature monitoring and compliance with GDP/GMP-related requirements (1, 2).

Digital transformation in healthcare supply chains involves the integration of data-driven processes, digital platforms and intelligent logistics practices. Recent reviews describe digital transformation as a mechanism for improving efficiency, transparency and responsiveness in healthcare supply chains (7, 8). From an ecosystem perspective, digital transformation also requires coordination between multiple stakeholders, technologies and institutional actors, as well as compliance with regulatory requirements and appropriate certification procedures by regulatory authorities (9).

In this review, digital transformation refers to the broader integration of data-driven processes, digital platforms and connected infrastructures, whereas supply chain optimization refers to specific improvements in forecasting, inventory management, monitoring and operational decision-making.

Emerging technologies are discussed in the literature as supporting multiple supply chain functions, including procurement, inventory management, transportation monitoring, pharmaceutical traceability and healthcare data integration. Blockchain is frequently discussed in relation to secure data sharing, traceability and anti-counterfeiting mechanisms in healthcare and pharmaceutical supply chains (10–12). In pharmaceutical cold chains, blockchain-enabled systems have also been explored for transparency and implementation challenges (13). In parallel, data analytics, artificial intelligence and machine learning are increasingly discussed in relation to demand forecasting, supply planning, inventory optimization, disruption management and operational decision support in pharmaceutical and healthcare supply chains (14–16). The convergence of blockchain, AI, IoT and cloud-based systems is increasingly examined as a possible pathway toward more interconnected and intelligent healthcare logistics models.

Although publications on digital technologies in healthcare and pharmaceutical supply chains have increased rapidly in recent years, the literature remains fragmented. Many studies focus on individual technologies, such as blockchain, artificial intelligence or IoT systems, or on specific applications, including pharmaceutical traceability, vaccine logistics or healthcare analytics. Fewer studies provide an integrated overview of how these technologies interact within broader healthcare logistics ecosystems. Therefore, an updated bibliometric mapping review is needed to identify dominant research themes, emerging technologies, geographic research distribution, technology-application relationships, temporal thematic evolution and implementation barriers across healthcare and pharmaceutical logistics research. Recent systematic and bibliometric reviews have mapped blockchain applications in the health supply chain, Industry 5.0 and healthcare supply-chain research, healthcare logistics, and the broader landscape of healthcare supply-chain management using an NLP-driven systematic review approach (17–20).

This study contributes to the literature by providing an integrated PRISMA-guided bibliometric mapping review of digital transformation across healthcare and pharmaceutical supply-chain contexts. Unlike technology-specific reviews that focus mainly on blockchain, artificial intelligence, IoT, vaccine logistics or healthcare analytics as separate domains, this review maps multiple digital and emerging technologies within a unified bibliometric corpus. The study combines annual production analysis, source and citation analysis, country-level scientific production, keyword co-occurrence mapping, temporal thematic evolution, technology-application relationships, implementation barriers and future research directions. Therefore, the contribution is not limited to descriptive publication counts, but provides an integrated overview of how digital technologies are represented, connected and interpreted within the healthcare and pharmaceutical supply-chain literature.

The aim of this study is to analyze the scientific landscape of digital transformation and emerging technologies in healthcare and pharmaceutical supply chains through bibliometric mapping and thematic interpretation.

The study was guided by the following research questions:

RQ1. How has research on digital transformation in healthcare and pharmaceutical supply chains evolved over time?

RQ2. Which technologies, journals, authors and countries dominate the field?

RQ3. What thematic clusters emerge from keyword co-occurrence analysis?

RQ4. How did thematic priorities evolve across pre-COVID, COVID and post-COVID periods?

RQ5. What implementation barriers and future research directions emerge from the literature?

## Materials and methods

This study was revised and structured as a PRISMA-guided bibliometric mapping review in accordance with the article-type requirements for Systematic Review submissions. The review systematically identified, screened, categorized and analyzed previous research on digital transformation and emerging technologies in healthcare and pharmaceutical supply chains. The methodological workflow included review question formulation, PICOS-informed framing, protocol clarification, database searching, eligibility screening, data extraction, bibliometric mapping, thematic synthesis and assessment of bibliometric quality considerations.

### Study design

This study employed a PRISMA-guided bibliometric mapping review design integrating systematic evidence identification, science mapping techniques and thematic interpretation. The review was designed to identify publication trends, influential sources, country-level scientific production, citation patterns, keyword co-occurrence structures, thematic clusters, temporal evolution and implementation barriers in digital healthcare and pharmaceutical supply chain research (21).

### PICOS-informed review framework

Because this study is a bibliometric mapping review rather than an intervention-effect review, the research question was adapted using a PICOS-informed framework. The population/context was defined as healthcare and pharmaceutical supply chains. The intervention/exposure included digital transformation and emerging technologies such as blockchain, artificial intelligence, Internet of Things, analytics, digital monitoring systems and data-driven healthcare logistics. No direct comparator was applied because the review aimed to map the research landscape rather than compare clinical or operational interventions. Outcomes included publication trends, influential sources, country-level research activity, citation patterns, keyword co-occurrence structures, thematic clusters, temporal research evolution, technology–application relationships, implementation barriers and future research directions. Eligible study designs included peer-reviewed journal articles indexed in Scopus, Web of Science and PubMed.

### Protocol and registration

No formal review protocol was registered. However, the review questions, search strategy, eligibility criteria, screening workflow, data extraction variables and bibliometric analysis procedures were defined before final analysis. The study selection process followed PRISMA 2020 principles and is reported using a PRISMA flow diagram.

### Data sources and search strategy

Scientific publications were retrieved from Scopus, Web of Science and PubMed databases. Searches were conducted on April 26, 2026 and covered publications published between January 2015 and April 2026. The search strategy combined terms related to healthcare supply chains, pharmaceutical logistics, blockchain, artificial intelligence, Internet of Things, digital transformation, healthcare technologies and supply chain management. Searches were restricted to English-language publications. Only peer-reviewed journal articles were included in the final quantitative bibliometric corpus. Conference proceedings, book chapters, editorials, notes and non-peer-reviewed publications were excluded from bibliometric mapping procedures to improve methodological consistency and comparability of bibliometric indicators. However, several highly relevant methodological and conceptual sources were selectively retained in the broader reference list to support interpretation and discussion.

The full search strategy used for Scopus, Web of Science and PubMed, including database-specific search strings, search fields, filters and search date, is provided in Supplementary Table S1. The final list of 83 included records used for bibliometric mapping is provided in Supplementary Dataset S2, allowing verification of the final analytical corpus and the reported selection pathway from 438 identified records to 147 records after duplicate removal, 105 reports assessed for eligibility and 83 included records.

Scopus, Web of Science and PubMed were selected because they provide broad multidisciplinary and biomedical coverage relevant to healthcare logistics, pharmaceutical supply chains and digital health technologies. However, the use of these three databases may have excluded relevant publications indexed in engineering-focused, computer science, regional or grey-literature databases.

A high duplicate rate was observed due to overlap across Scopus, PubMed and Web of Science indexing systems. Duplicate removal was conducted using DOI matching, title comparison and manual verification procedures. Potential duplicates and records with missing or inconsistent DOI information were manually checked by comparing titles, authors, publication years and source information. Table 1 summarizes the databases, search fields, filters and export formats used during data retrieval and harmonization.

**Table 1:** Database retrieval and harmonization.

Database	Search Field	Filters	Retrieved	Export Format
Scopus	TITLE-ABS-KEY	English, journal articles	131	CSV
Web of Science	Topic Search (TS)	English, journal articles	79	TXT
PubMed	Title/Abstract	English, journal articles	228	NBIB
Total			438	

### Dataset reconciliation and duplicate removal

Search results from Scopus, Web of Science and PubMed were exported separately and harmonized before bibliometric analysis. The initial number of retrieved records represented raw database-specific search outputs. Duplicate removal was performed in two stages. First, records were screened using DOI matching and title comparison. Second, potentially duplicated records and records with missing or inconsistent DOI information were manually verified by comparing title, authors, publication year and source information. Records were considered duplicates when they had the same DOI or, when DOI information was missing, highly similar title, author and publication-year information. Because database indexing, export formats and document-type classifications differed across Scopus, Web of Science and PubMed, harmonization was performed before final eligibility screening. After duplicate removal and harmonization, 147 unique records remained for title and abstract screening.

### Eligibility criteria

Studies were included if they:

- addressed digital technologies in healthcare or pharmaceutical supply chains;
- focused on blockchain, AI, IoT, analytics, or digital transformation;
- examined logistics, traceability, inventory management, or healthcare distribution systems;
- were published as peer-reviewed journal articles in English.

Studies were excluded if they:

- were unrelated to healthcare or pharmaceutical supply chains;
- represented conference proceedings or book chapters;
- lacked sufficient methodological detail;
- focused on unrelated industries.

### Study selection and data extraction

The study selection process was conducted in accordance with PRISMA 2020 principles. A total of 438 records were identified across Scopus, PubMed and Web of Science. After duplicate removal, 147 records remained for title and abstract screening.

During screening, 42 records were excluded because they were not directly related to healthcare or pharmaceutical supply chains or did not meet the document-type criteria. Subsequently, 105 reports were assessed for eligibility through full-text evaluation.

An additional 22 reports were excluded due to insufficient methodological detail, lack of direct supply-chain focus or inaccessible full-text content. As a result, 83 peer-reviewed journal articles were retained for the final bibliometric mapping review. Bibliographic metadata were extracted from the final corpus, including article titles, authors, publication years, journals, abstracts, author keywords, citations, DOI information and available affiliation metadata. The final bibliometric corpus is provided as Supplementary Dataset S2. This dataset contains the 83 included records used for annual scientific production analysis, source analysis, citation analysis, keyword frequency analysis, keyword co-occurrence mapping, temporal thematic evolution and technology–application interpretation. Providing this dataset enables verification of the final corpus and improves reproducibility of the bibliometric results. Title/abstract screening was initially performed by the corresponding author and subsequently checked by a second author for consistency with the predefined eligibility criteria. Full-text eligibility decisions and uncertain cases were discussed among the authors and resolved by consensus. «Insufficient methodological detail» was defined as the absence of sufficient information to determine the study design, data source, analytical approach or relevance to bibliometric and thematic extraction. «No direct healthcare or pharmaceutical supply-chain focus» was defined as studies addressing digital technologies or healthcare systems generally without a clear connection to logistics, distribution, inventory management, traceability, cold-chain monitoring or pharmaceutical supply-chain processes. «Full-text content unavailable for eligibility verification» referred to records for which the full text could not be accessed after reasonable retrieval attempts.

**Figure 1:**
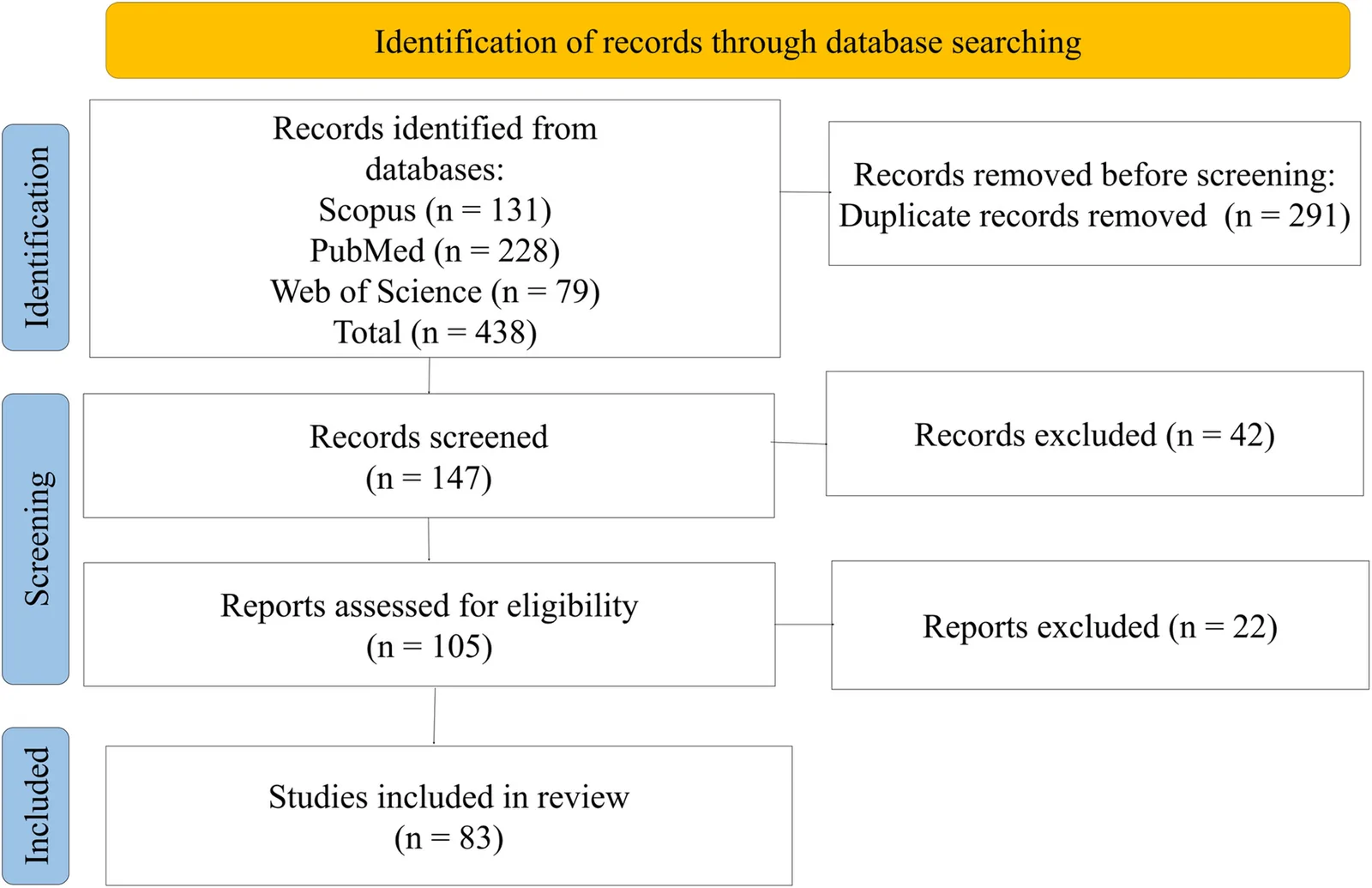
PRISMA-based study selection process for the bibliometric review.

Table 2 summarizes the exclusion reasons according to the PRISMA selection stage. Exclusions made during title/abstract and document-type screening are reported separately from exclusions made during full-text eligibility assessment to ensure consistency with the PRISMA flow diagram (Figure 1). Because document-type classifications differed across databases, conference proceedings and book chapters were identified during harmonization and excluded before final bibliometric mapping.

**Table 2:** Exclusion reasons during screening and full-text eligibility assessment.

Selection stage	PRISMA unit	Reason for exclusion	Number
Title/abstract and document-type screening	Records	Not directly related to healthcare or pharmaceutical supply chains	11
Title/abstract and document-type screening	Records	Conference proceedings	17
Title/abstract and document-type screening	Records	Book chapters	14
**Subtotal**	**Records**	**Records excluded during screening**	**42**
Full-text eligibility assessment	Reports	Insufficient methodological detail	6
Full-text eligibility assessment	Reports	No direct healthcare or pharmaceutical supply-chain focus after full-text evaluation	5
Full-text eligibility assessment	Reports	Full-text content unavailable for eligibility verification	4
Full-text eligibility assessment	Reports	Other final eligibility criteria not met	7
**Subtotal**	**Reports**	**Reports excluded after full-text eligibility assessment**	**22**

Note. Records refer to items screened after duplicate removal. Reports refer to full-text publications considered for eligibility. The subtotals correspond to the PRISMA flow diagram: 42 records were excluded during title/abstract and document-type screening, and 22 reports were excluded after full-text eligibility assessment.

Bold values indicate subtotal rows corresponding to PRISMA stage-level totals.

### Data analysis and bibliometric mapping

Bibliometric mapping and conceptual structure analyses were performed using VOSviewer version 1.6.20 and Bibliometrix/Biblioshiny version 5.3.0 in R version 4.5.2 (22, 23). Additional customized visualizations were generated using Python version 3.12.13 with pandas version 2.2.2 and matplotlib version 3.10.0. The analysis included annual scientific production, source analysis, citation analysis, country-level scientific production, keyword frequency analysis, keyword co-occurrence mapping, thematic clustering and temporal thematic evolution. Standardized author keywords were used as the primary unit of keyword co-occurrence analysis. In the final revised analysis, keyword co-occurrence mapping was conducted in VOSviewer using the full counting method, with a minimum keyword occurrence threshold of 2 occurrences. This threshold was selected because the final bibliometric corpus was relatively small (*n* = 83), and a lower threshold allowed the analysis to retain both dominant and emerging terms relevant to digital transformation in healthcare and pharmaceutical supply chains. Normalization was performed using the VOSviewer association-strength method, and clustering was conducted using the default VOSviewer clustering settings. Before analysis, keywords were manually standardized using a thesaurus/manual harmonization procedure to merge synonyms, abbreviations, spelling variants, capitalization differences and duplicate terms, including variants such as «AI»/«artificial intelligence», «IoT»/«Internet of Things», «COVID-19»/«Covid 19» and singular/plural supply-chain terms.

To improve methodological transparency and readability, Table 3 summarizes the main bibliometric analysis procedures, data fields, analytical tools, outputs and interpretation purposes used in this review.

**Table 3:** Bibliometric analysis procedures, data fields, tools and outputs.

Analytical component	Data field/unit of analysis	Tool/procedure	Output	Interpretation
Annual scientific production	Publication year	Biblioshiny/Python visualization	Annual publication trend	Identifies temporal growth of research activity
Source analysis	Journal titles	Bibliometrix/frequency analysis	Most productive sources	Shows disciplinary distribution of publications
Citation analysis	Citation counts and publication metadata	Bibliometrix/manual verification	Most influential publications	Identifies highly cited studies within the corpus
Country-level production	Author affiliation metadata	Bibliometrix/mapping visualization	Geographic distribution map	Shows regional distribution of indexed research activity
Keyword frequency analysis	Author keywords	Keyword standardization and frequency analysis	Most frequent keywords	Identifies dominant technological and conceptual terms
Keyword co-occurrence mapping	Standardized author keywords	VOSviewer	Co-occurrence network	Identifies conceptual associations between research themes
Temporal thematic evolution	Keywords by publication period	Biblioshiny/Python heatmap	Pre-COVID, COVID and post-COVID thematic trends	Shows changes in research attention over time
Technology–application mapping	Technologies and application areas identified in the literature	Thematic interpretation	Technology–application diagram	Summarizes literature-based associations, not causal effects
Implementation barrier synthesis	Extracted barriers from included studies	Thematic synthesis	Barrier table	Identifies implementation challenges discussed in the literature

This analytical workflow was designed to combine quantitative bibliometric mapping with qualitative thematic interpretation. Therefore, the analysis identifies publication patterns, conceptual structures and literature-based technology–application associations, but does not evaluate the clinical or operational effectiveness of the reviewed technologies. Manual keyword standardization was used to merge synonyms and abbreviations, but residual terminology variation may remain and was considered when interpreting the thematic network.

For temporal interpretation, publications were grouped into three descriptive periods: pre-COVID period (2015–2019), COVID period (2020–2021), and post-COVID period (2022–April 2026). These periods were used only to compare thematic visibility over time and were not interpreted as causal intervention periods. Because the database search/export cutoff date was April 26, 2026, records from 2026 were treated as an incomplete publication year. Citation counts reported in Table 4 were extracted from the total-citation field available in the final merged bibliographic metadata derived from the Scopus, Web of Science and PubMed exports using the same cutoff date. Citation rankings were based on absolute citation counts and were not normalized by publication year.

**Table 4:** Most influential publications in digital healthcare and pharmaceutical supply chains.

Title	Authors	Journal	Year	Total citations
Blockchain for Industry 4.0: A comprehensive review	Bodkhe et al. (32)	IEEE Access	2020	689
A review of existing and emerging digital technologies to combat the global trade in fake medicines	Mackey & Nayyar (1)	Expert Opinion on Drug Safety	2017	298
Leveraging digital capabilities toward a circular economy: Reinforcing sustainable supply chain management with Industry 4.0 technologies	Liu et al. (33)	Computers and Industrial Engineering	2023	278
Pharma Industry 4.0: Literature review and research opportunities in sustainable pharmaceutical supply chains	Ding (34)	Process Safety and Environmental Protection	2018	219
Roles of innovation leadership on using Big Data Analytics to establish resilient healthcare supply chains to combat the COVID-19 pandemic: A multimethodological study	Bag et al. (25)	IEEE transactions on engineering management	2024	168
Big data analytics and artificial intelligence technologies based collaborative platform empowering absorptive capacity in health care supply chain: An empirical study	Bag et al. (15)	Journal of Business Research	2023	166
Vaccine supply chain management: An intelligent system utilizing blockchain, IoT and machine learning	Hu et al. (76)	Journal of Business Research	2023	150
The healthcare sustainable supply chain 4.0: The circular economy transition conceptual framework with the corporate social responsibility mirror	Daú et al. (35)	Sustainability	2019	147

### Assessment of risk of bias and quality considerations

Traditional clinical risk-of-bias assessment was not applied because this study did not synthesize intervention effects, clinical outcomes or comparative evidence. Instead, quality considerations focused on bibliometric and mapping-review risks, including database coverage, English-language restriction, citation-age effects, citation visibility, missing or inconsistent metadata, variability in author keyword usage and interpretation bias. These issues were addressed through transparent database selection, predefined eligibility criteria, DOI and title-based duplicate removal, manual verification, keyword standardization and PRISMA-based reporting. Remaining limitations are discussed in the Limitations section.

## Results

The final PRISMA-guided bibliometric mapping review included 83 peer-reviewed journal articles published between 2015 and 2026. The results below report study selection and corpus characteristics, publication trends, influential sources, geographic research distribution, citation patterns, thematic structures and implementation-related findings.

### Annual scientific production

**Figure 2:**
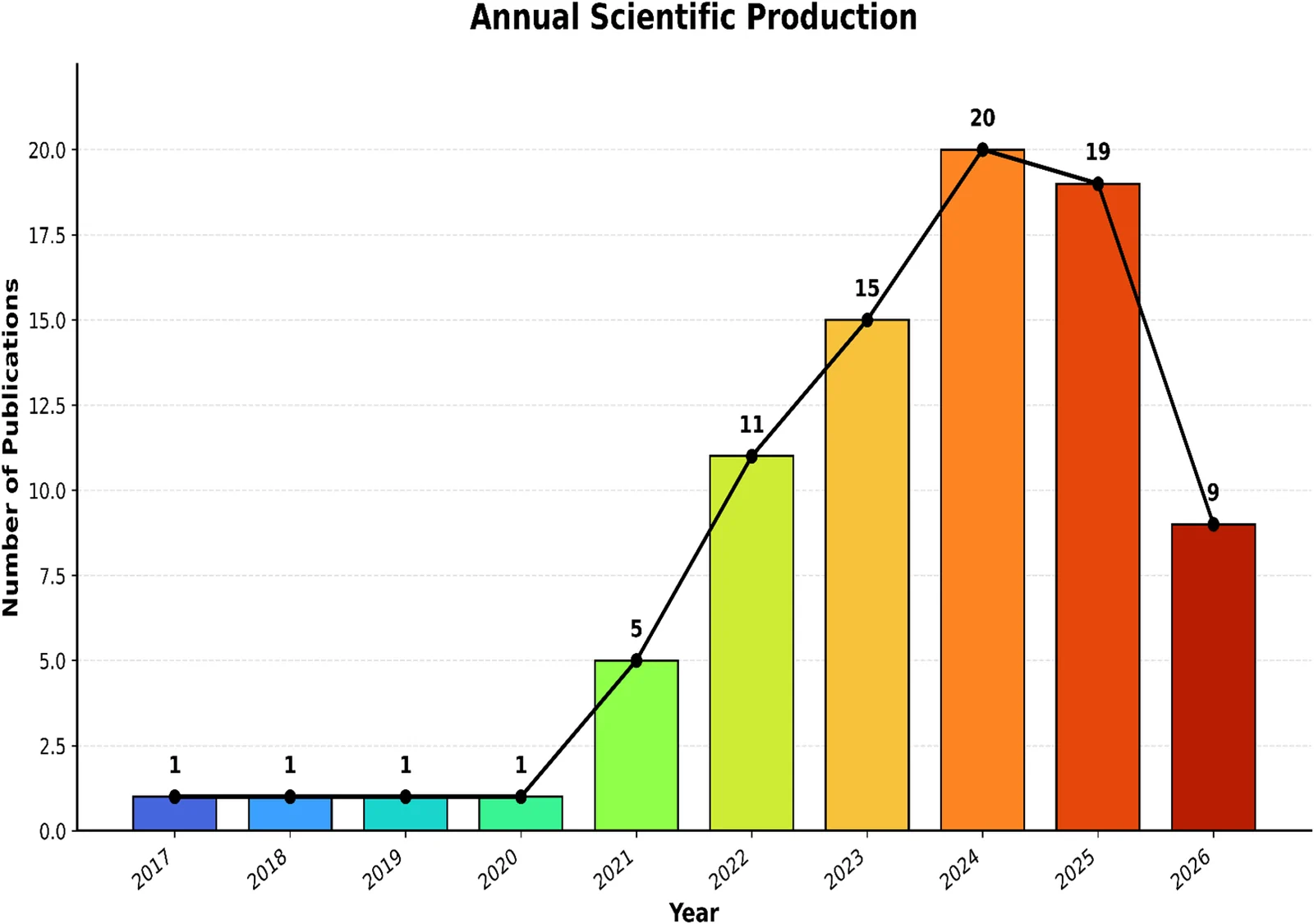
Annual scientific production in digital transformation of healthcare and pharmaceutical supply chains in the final bibliometric corpus (*n* = 83). Data for 2026 include records indexed up to April 26, 2026.

Figure 2 presents the annual scientific production within the final bibliometric corpus of 83 publications. Publication activity remained limited from 2017 to 2020, with one publication per year. A clear increase was observed from 2021 onward, rising from 5 publications in 2021 to 11 in 2022, 15 in 2023 and reaching the highest level in 2024 with 20 publications. A similarly high level was observed in 2025 with 19 publications, while 2026 included 9 publications because the search covered records indexed up to April 26, 2026. Overall, the distribution indicates growing research attention to digital transformation, emerging technologies and resilient healthcare and pharmaceutical supply chains after 2020. This interpretation is consistent with broader studies describing the movement from isolated digital applications toward more integrated and resilient digital healthcare supply chain ecosystems (24–27). Related studies have further examined digital technologies in logistics, supply-chain visibility and resilience in healthcare supply chains, blockchain/IoT applications in the pharmaceutical sector, and blockchain-based security and privacy in smart healthcare, supporting the broader interpretation of increasing digital-technology attention across logistics, healthcare and pharmaceutical contexts (28–31).

### Most relevant sources and authors

The bibliometric analysis identified several productive publication sources and contributing authors within the research field of digital transformation in healthcare and pharmaceutical supply chains. Journals specializing in healthcare analytics, logistics, digital transformation, operations management and intelligent supply chain systems demonstrated the highest publication activity within the analyzed corpus.

**Figure 3:**
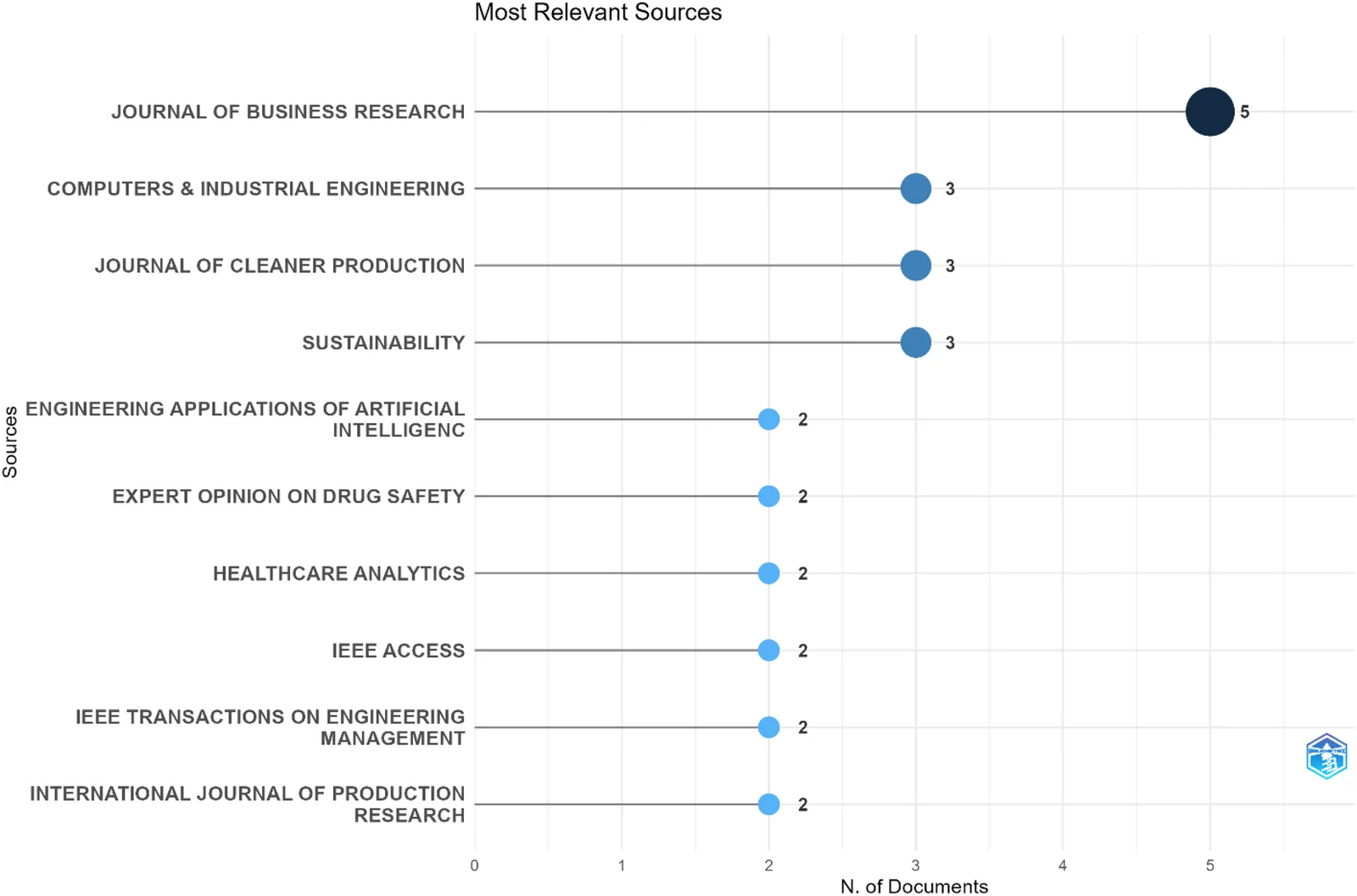
Most productive publication sources in digital healthcare and pharmaceutical supply chain research.

The source analysis shows that research in this domain is distributed across interdisciplinary journals integrating healthcare management, digital technologies, logistics, engineering and supply chain analytics. As shown in Figure 3, the Journal of Business Research was the most productive publication source within the analyzed corpus, followed by Sustainability, Journal of Cleaner Production and Computers & Industrial Engineering. Additional contributions were identified in journals focusing on healthcare analytics, artificial intelligence, engineering management and pharmaceutical logistics.

The distribution of publications across multiple disciplinary domains supports the interpretation of the interdisciplinary structure of the field, combining healthcare systems research, pharmaceutical logistics, intelligent technologies and operational resilience studies.

The authorship structure remained relatively dispersed, indicating that the field is still evolving and characterized by contributions from diverse research communities, including healthcare management, logistics, engineering, information systems and digital transformation research.

### Global research distribution and country scientific production

**Figure 4:**
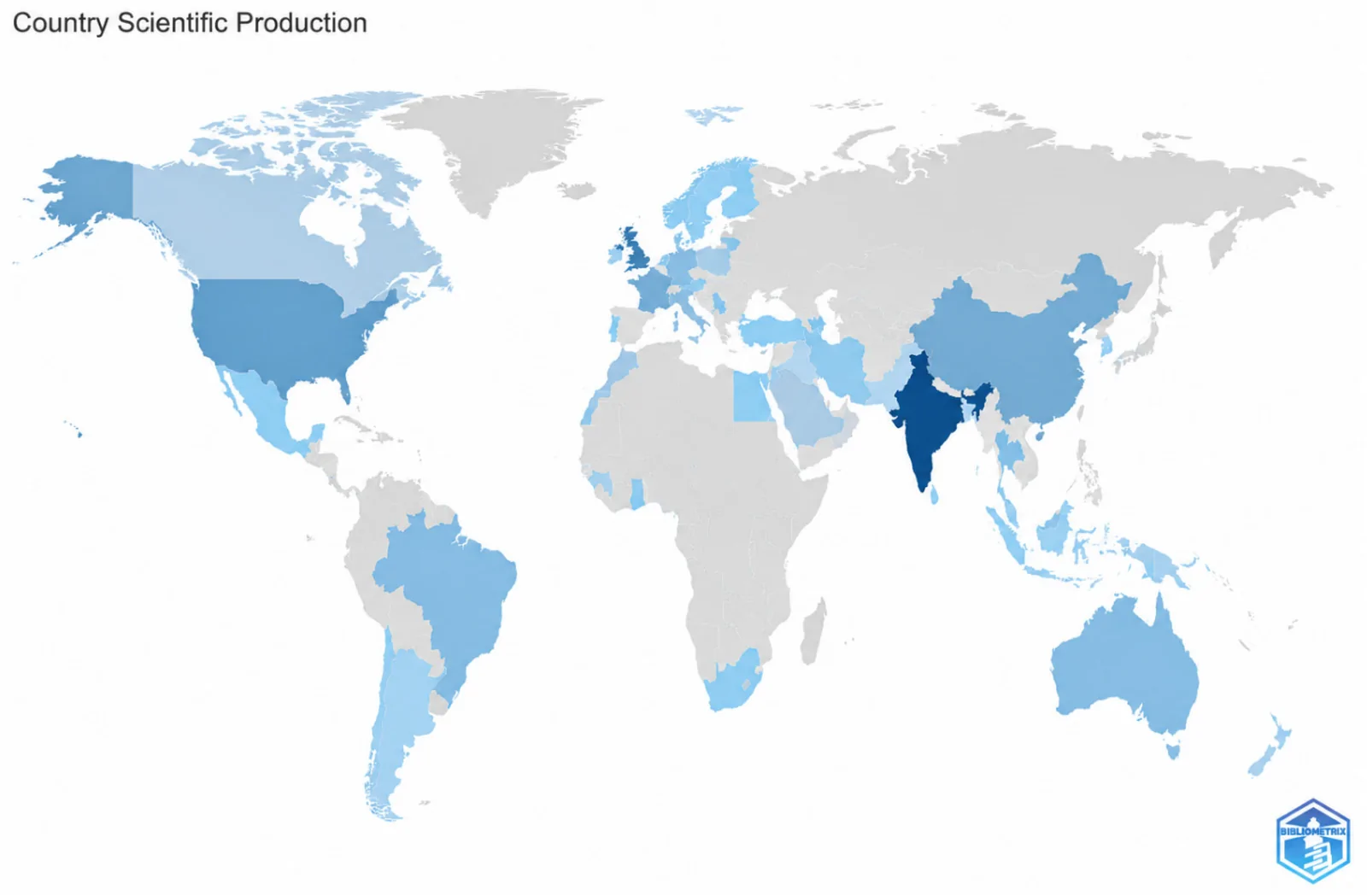
Global distribution of scientific production in digital healthcare and pharmaceutical supply chain research.

The country-level analysis shows that interest in digital transformation of healthcare and pharmaceutical supply chains has expanded across multiple regions worldwide. Research activity was particularly visible in Asia, North America and Europe, indicating that digital healthcare logistics and pharmaceutical traceability have become globally relevant research directions (Figure 4).

India demonstrated the highest publication activity within the analyzed dataset, which may reflect the country's growing focus on healthcare digitalization, pharmaceutical manufacturing and supply chain modernization. Strong contributions were also identified from the United States, China, the United Kingdom and several European countries. These countries are actively involved in research related to blockchain-based traceability, artificial intelligence, healthcare analytics and resilient healthcare logistics systems.

The map also illustrates that research in this field is not limited to one geographic region or discipline. Instead, it combines expertise from healthcare management, engineering, logistics, information systems and pharmaceutical sciences, highlighting the multidisciplinary nature of digital healthcare supply chain research.

This geographic distribution should be interpreted as publication activity within the analyzed corpus and may be influenced by database coverage and English-language indexing.

### Influential publications and citation trends

Citation analysis showed that blockchain-related publications had the highest citation visibility within the analyzed corpus, particularly studies focusing on traceability, transparency and counterfeit prevention in pharmaceutical supply chains. This citation pattern should be interpreted as bibliometric visibility rather than direct evidence of practical impact.

Studies addressing AI-driven optimization, healthcare resilience and vaccine supply chain management also demonstrated strong citation performance, particularly after the COVID-19 pandemic. However, citation counts may be influenced by publication age, journal visibility, thematic popularity and database coverage.

Citation analysis identified several highly visible publications that shaped the development of digital transformation research in healthcare and pharmaceutical supply chains (Table 4). The most cited studies primarily focus on blockchain technologies, pharmaceutical traceability, counterfeit prevention and Industry 4.0 applications in healthcare logistics. Table 4 reports absolute citation counts; therefore, the ranking provides visibility within the corpus but does not normalize citation impact by publication year.

The results also demonstrate the interdisciplinary nature of the field, integrating healthcare management, supply chain analytics, digital technologies and operational resilience frameworks.

### Keyword frequency analysis and dominant research themes

**Figure 5:**
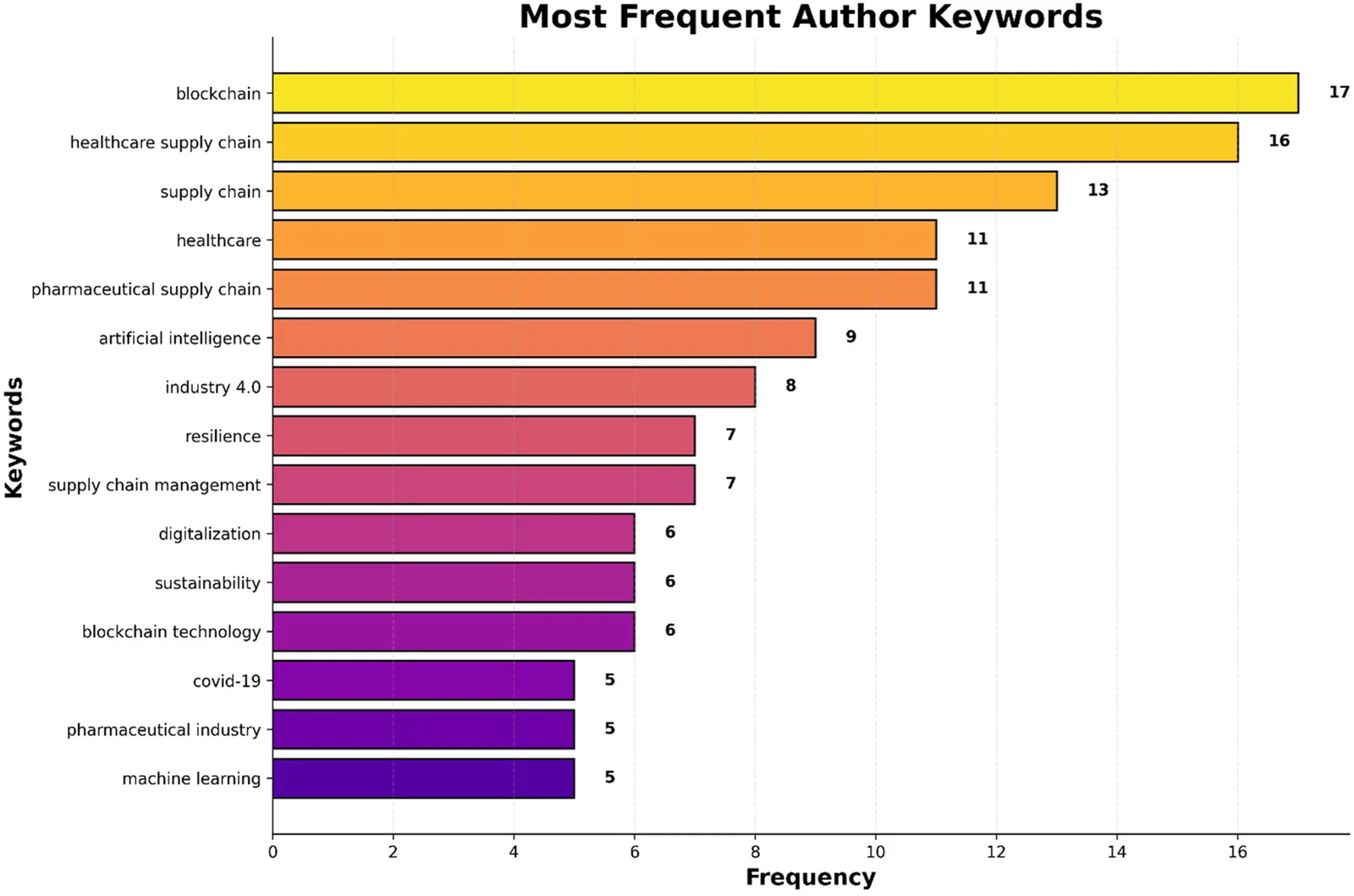
Most frequent author keywords.

Keyword frequency analysis was performed to identify dominant technological and conceptual themes within the final bibliometric dataset. As shown in Figure 5, blockchain was the most frequently occurring keyword, followed by healthcare supply chain, pharmaceutical supply chain, artificial intelligence and Industry 4.0. Keyword frequency analysis identifies dominant terms within the corpus, but emerging themes with lower frequency may be underrepresented. Therefore, frequency results were interpreted together with co-occurrence mapping and temporal thematic evolution. The visibility of blockchain-related terminology reflects strong research attention to pharmaceutical traceability, transparency and counterfeit prevention. This dominance reflects keyword frequency and citation visibility within the analyzed literature and should not be interpreted as evidence that blockchain is more effective than other digital technologies in practice. At the same time, the growing presence of artificial intelligence, machine learning and digitalization-related keywords reflects increasing scientific interest in predictive analytics, intelligent logistics systems and data-driven healthcare operations.

Keywords associated with resilience, COVID-19 and cold chain monitoring also appeared frequently, suggesting increased research attention to digitally supported healthcare logistics during and after the pandemic period. The keyword distribution suggests that the field is structured around three major thematic directions: blockchain-enabled transparency and traceability, digital transformation of healthcare and pharmaceutical logistics, and intelligent supply chain optimization through artificial intelligence, machine learning and analytics. Thematic clusters were generated through keyword co-occurrence mapping and then manually interpreted to clarify the conceptual structure of the field.

### Temporal evolution of research themes

**Figure 6:**
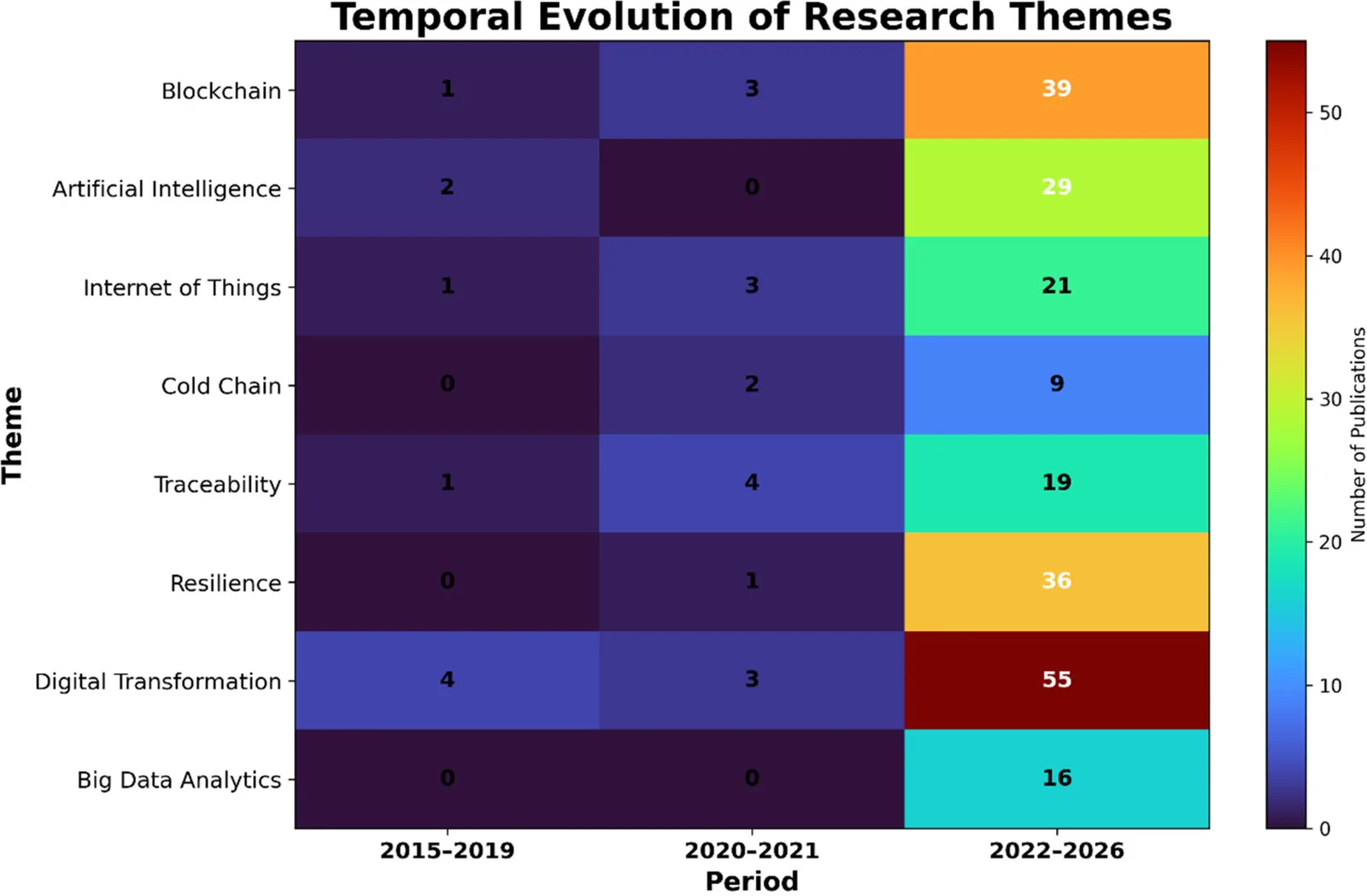
Temporal evolution of research themes across pre-COVID, COVID and post-COVID periods.

The temporal evolution analysis describes how research priorities changed across the pre-pandemic, pandemic and post-pandemic periods (Figure 6). These periods were used as descriptive analytical categories to compare thematic visibility over time rather than to establish causal effects. During 2015–2019, the literature was relatively limited and mainly focused on blockchain technologies, pharmaceutical traceability and early digital healthcare applications. Research activity increased during 2020–2021, particularly in areas related to vaccine logistics, cold chain monitoring and supply chain resilience, indicating a bibliometric shift in research attention during this period. The COVID-19 pandemic highlighted vulnerabilities in healthcare logistics systems and may have contributed to increased interest in technologies associated with operational visibility and real-time monitoring. The post-pandemic period (2022–2026) showed the strongest thematic expansion. Digital transformation, blockchain, resilience and artificial intelligence became the most visible research directions, suggesting increased research attention to integrated and technology-enabled healthcare supply chain models. The growing presence of IoT and predictive analytics themes further indicates increasing interest in intelligent healthcare logistics and connected monitoring infrastructures.

The heatmap shows a clear expansion of research activity in the post-COVID period. During 2015–2019, the field remained relatively limited, with early attention to digital transformation, artificial intelligence, blockchain and traceability. During 2020–2021, research activity increased moderately, particularly around blockchain, Internet of Things, cold chain and traceability themes, reflecting the growing relevance of vaccine logistics and digitally supported monitoring systems during the COVID-19 pandemic. The most substantial increase occurred during 2022–2026. Digital transformation became the most prominent theme (*n* = 55), followed by blockchain (*n* = 39), resilience (*n* = 36), artificial intelligence (*n* = 29), Internet of Things (*n* = 21) and traceability (*n* = 19). These results may indicate a transition in research attention from isolated technology applications toward integrated digital healthcare supply chain ecosystems. The strong growth of resilience and digital transformation themes also suggests that recent research increasingly focuses not only on technological adoption but also on the ability of healthcare and pharmaceutical supply chains to respond to disruption, uncertainty and operational complexity.

### Three-Fields analysis: keywords, authors and journals

**Figure 7:**
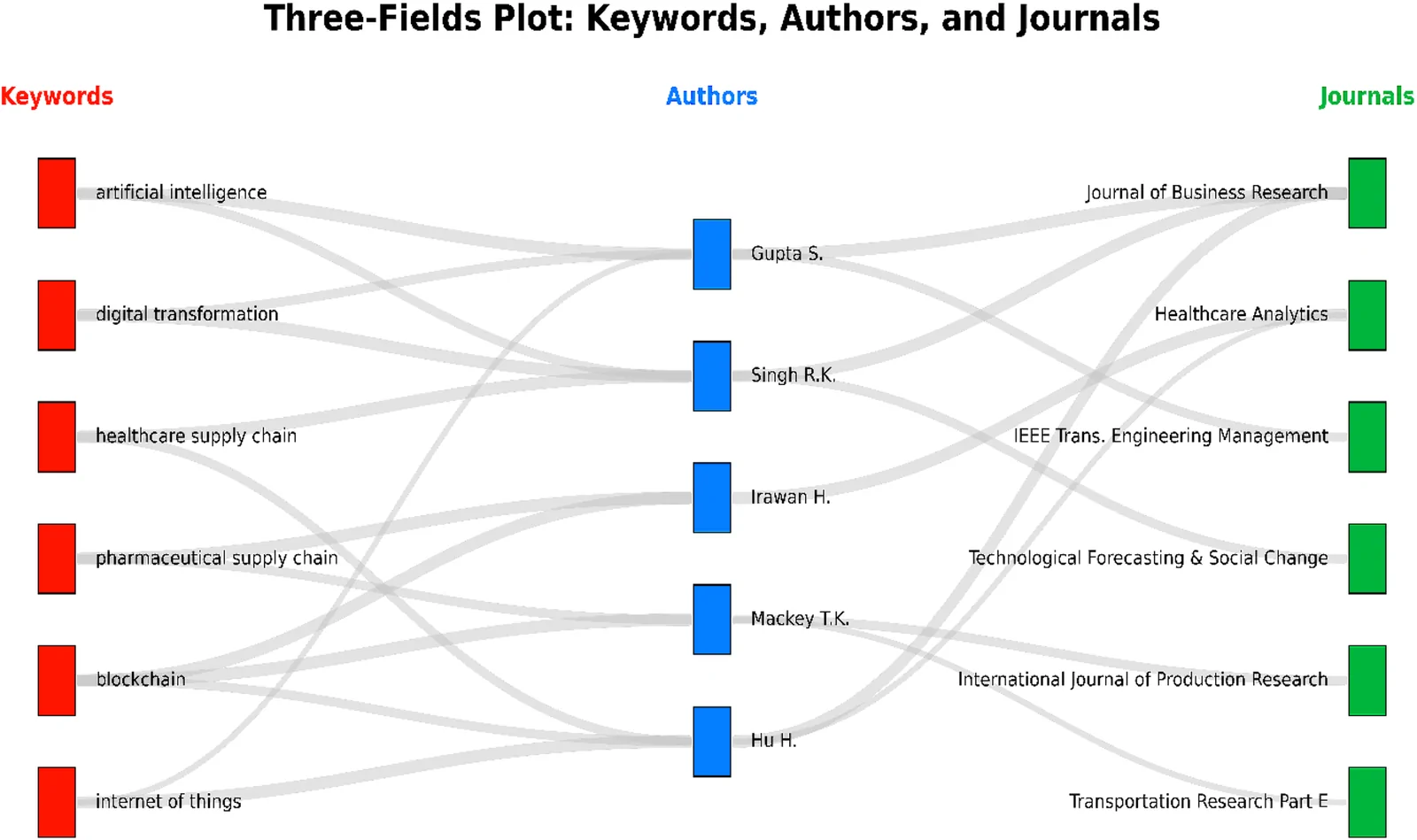
Three-fields plot linking dominant keywords, productive authors, and leading journals.

A three-fields plot was developed to visualize the relationships between dominant keywords, productive authors and leading journals in the analyzed corpus. This type of visualization helps clarify how key research themes are connected with influential contributors and publication sources. Figure 7 links the main thematic terms, including artificial intelligence, digital transformation, healthcare supply chain, pharmaceutical supply chain, blockchain, and Internet of Things, with selected authors and major journals in the field. This pattern is consistent with previous research showing that digital transformation in healthcare and pharmaceutical supply chains is distributed across technology, logistics, management and healthcare-oriented research domains (36).

The three-fields plot supports the interpretation of the interdisciplinary character of research on digital transformation in healthcare and pharmaceutical supply chains. The left side of the figure shows the dominant thematic areas, while the middle and right sides show their connections with authors and journals. Artificial intelligence, blockchain, digital transformation, healthcare supply chain and pharmaceutical supply chain are strongly connected with authors contributing to healthcare logistics, supply chain analytics and technology-enabled supply chain management.

The journal distribution further shows that the field is not concentrated in one disciplinary area. Instead, it spans business research, healthcare analytics, engineering management, production research, technology forecasting and transportation logistics.

### Keyword co-occurrence network

**Figure 8:**
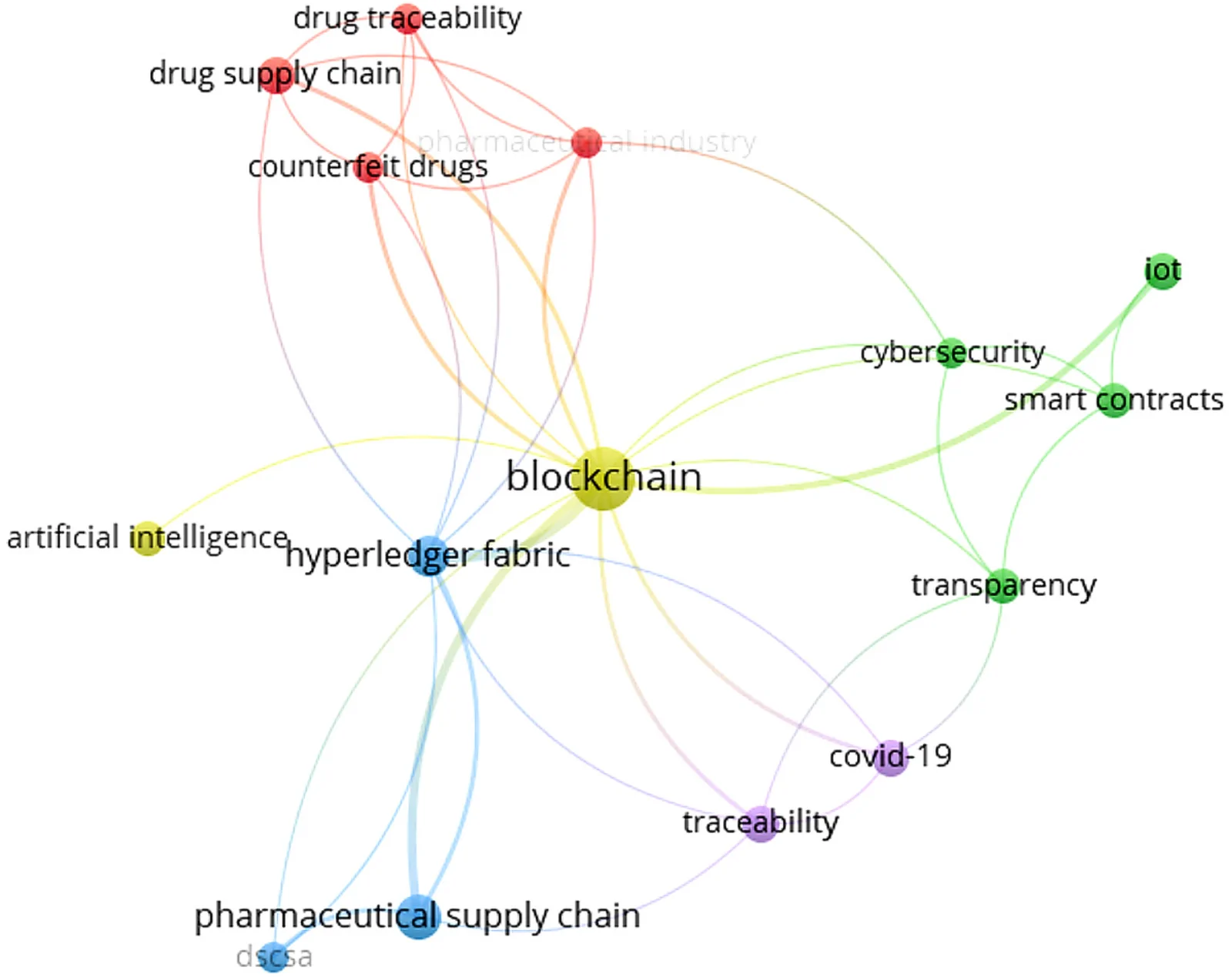
Keyword co-occurrence network of digital transformation in healthcare and pharmaceutical supply chains.

The keyword co-occurrence network provides a broader view of conceptual associations among author keywords within the analyzed literature (Figure 8). Blockchain appeared as the most visible connecting theme, linking pharmaceutical traceability, transparency, cybersecurity, smart contracts and healthcare logistics systems. These links should be interpreted as keyword co-occurrence patterns rather than causal or operational relationships. Several interconnected thematic clusters were identified within the network. One major cluster focused on pharmaceutical security and counterfeit prevention, highlighting growing research attention to secure medicine distribution and regulatory traceability systems. Another cluster emphasized IoT technologies, connected monitoring systems and data security, reflecting increasing interest in digitally integrated healthcare infrastructures. Artificial intelligence and machine learning appeared as emerging research directions associated with forecasting, optimization and decision-support applications in healthcare logistics.

The appearance of DSCSA (Drug Supply Chain Security Act) within the thematic network was interpreted as one example of regulatory traceability frameworks discussed in the literature, rather than as a globally representative or dominant regulatory model. Similar themes are emphasized in studies examining blockchain-enabled pharmaceutical traceability and supply-chain visibility systems (1–3). The network visualization was refined through manual keyword screening to improve thematic specificity and interpretability.

The thematic clusters identified through keyword co-occurrence analysis were further interpreted to clarify the conceptual structure of the research field. Table 5 summarizes the major thematic areas emerging from the co-occurrence network.

**Table 5:** Interpreted thematic clusters identified through keyword co-occurrence analysis.

Cluster	Core keywords	Interpretive summary
Blockchain-enabled traceability	blockchain, traceability, smart contracts, transparency	Focus on secure pharmaceutical traceability, anti-counterfeit systems and trusted supply-chain visibility
Pharmaceutical security and compliance	counterfeit drugs, drug traceability, cybersecurity, DSCSA	Research on pharmaceutical authenticity verification, regulatory compliance and secure medicine distribution
Digital healthcare infrastructure	IoT, cybersecurity, transparency, data security	Connected monitoring systems and digital healthcare logistics infrastructures
Intelligent healthcare analytics	artificial intelligence, machine learning	Emerging use of intelligent systems for healthcare logistics optimization and predictive analytics
Pandemic resilience and disruption	COVID-19, resilience	Digital technologies supporting healthcare supply-chain resilience during disruptions and emergencies

The cluster structure demonstrates that blockchain-related traceability and pharmaceutical security themes dominate the research field, while artificial intelligence and IoT technologies represent emerging directions associated with intelligent analytics and connected healthcare infrastructures. The presence of COVID-19 and resilience-related terms further indicates increasing attention toward disruption management and adaptive healthcare logistics systems.

### Technology-Application relationships

**Figure 9:**
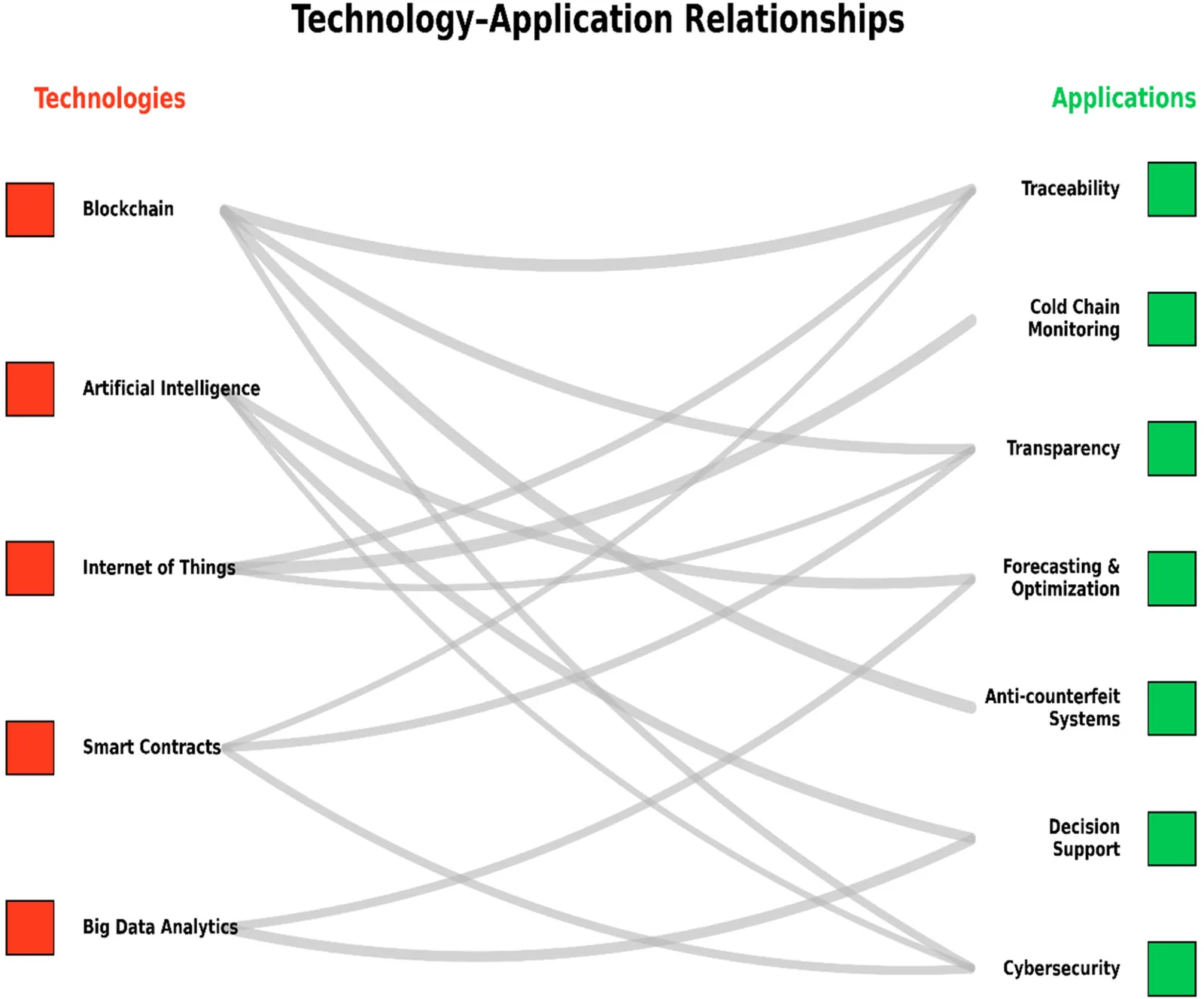
Technology-application relationships in digital healthcare and pharmaceutical supply chains.

To further interpret the literature-based associations between emerging technologies and application areas within healthcare and pharmaceutical supply chains, a technology–application relationship diagram was constructed. Figure 9 summarizes the primary technological domains and their most common applications identified across the reviewed literature.

These associations reflect how technologies are discussed in the literature and should not be interpreted as direct evidence of operational implementation or effectiveness. Blockchain technologies were most frequently associated with traceability, transparency, anti-counterfeit systems and cybersecurity applications. Internet of Things technologies were associated with sensor-based cold chain monitoring, temperature visibility and connected logistics infrastructures. Artificial intelligence and big data analytics were mainly associated with forecasting, optimization and decision-support functions in the reviewed literature. These technology–application patterns are consistent with studies discussing digital, intelligent and data-driven approaches to healthcare and pharmaceutical supply chain transformation (37–39).

### Implementation barriers and practical implications

Despite the growing scientific interest in digital transformation within healthcare and pharmaceutical supply chains, the reviewed literature identifies several major barriers limiting the large-scale implementation of emerging technologies. These barriers involve technological, organizational, regulatory, financial and workforce-related dimensions affecting the operational integration of blockchain, artificial intelligence, Internet of Things and analytics-driven systems.

The findings indicate that interoperability limitations remain one of the most frequently discussed challenges. Many healthcare and pharmaceutical systems continue to operate using fragmented digital infrastructures that complicate data exchange and integration across manufacturers, distributors, healthcare providers, regulators and logistics operators. The absence of standardized interoperability frameworks limits the scalability of digital healthcare ecosystems and reduces the effectiveness of traceability and monitoring systems. The major implementation barriers are summarized in Table 6.

**Table 6:** Implementation barriers in digital healthcare and pharmaceutical supply chains.

Barrier	Description	Practical implications
Interoperability limitations	Lack of standardized digital infrastructure	Difficult integration across healthcare stakeholders
Data privacy and cybersecurity concerns	Sensitive healthcare and logistics data	Increased security and compliance requirements
High implementation costs	Infrastructure and integration expenses	Limited adoption in resource-constrained systems
Regulatory complexity	GDP/GMP/GSP and traceability compliance requirements	Delayed implementation and validation processes
Technical expertise shortages	Limited digital workforce competencies	Reduced operational readiness and scalability
Scalability issues	Challenges integrating multiple stakeholders	Difficult expansion of digital ecosystems

Data privacy and cybersecurity concerns also represent major implementation barriers. Healthcare and pharmaceutical supply chains involve highly sensitive operational and patient-related information, requiring secure data-sharing architectures and compliance with regulatory data-protection requirements. Blockchain technologies, while improving transparency and traceability, also introduce challenges related to secure access control, governance and integration with existing digital infrastructures.

Financial constraints and implementation costs were frequently identified in the reviewed literature. The deployment of blockchain-enabled traceability systems, IoT-based cold chain monitoring infrastructures and AI-driven analytics platforms often requires substantial investments in digital infrastructure, software integration, hardware and workforce training. These financial barriers are particularly relevant in resource-constrained healthcare systems and developing pharmaceutical markets (40–43).

Regulatory complexity further complicates digital transformation initiatives in pharmaceutical logistics. International GDP/GMP/GSP requirements, pharmaceutical serialization regulations, traceability standards and validation procedures increase implementation complexity and may delay the adoption of new technologies. The Drug Supply Chain Security Act is discussed as one example of a regulatory traceability framework supporting secure pharmaceutical distribution and counterfeit prevention initiatives, rather than as a universal global standard (44, 45). Another recurring challenge involves shortages of technical expertise and digital competencies among healthcare and pharmaceutical personnel. The successful implementation of AI systems, blockchain infrastructures and IoT-enabled monitoring platforms requires interdisciplinary expertise combining healthcare operations, logistics management, information systems, cybersecurity and regulatory compliance knowledge.

The practical implications of these findings are significant for healthcare organizations, pharmaceutical manufacturers, distributors, regulators and policymakers. The reviewed literature suggests that blockchain technologies may support pharmaceutical traceability, anti-counterfeit protection and secure medicine distribution, while IoT-based systems are discussed as tools for real-time cold chain monitoring and connected logistics infrastructures (46, 47). Artificial intelligence and big data analytics are increasingly discussed as tools for forecasting, inventory optimization and decision support, although their operational value depends on data quality, implementation maturity and organizational readiness (48–51).

### Quality and bias considerations

Because this review focused on bibliometric mapping rather than clinical intervention effects, no traditional clinical risk-of-bias score was calculated. The main sources of potential bias were database coverage, English-language restriction, citation-age effects, citation visibility, keyword variability, incomplete metadata across databases and interpretation bias. These factors were considered when interpreting citation patterns, country-level production, thematic clusters and co-occurrence structures.

## Discussion

### Interpretation of major bibliometric trends

The findings should be interpreted within the boundaries of bibliometric evidence. Co-occurrence maps, citation patterns and thematic clusters identify patterns of research attention and conceptual association, but they do not establish causal relationships, implementation effectiveness or real-world performance of digital technologies.

The increase in publications after 2020 is consistent with heightened research attention during and after the COVID-19 pandemic. During this period, healthcare systems around the world faced major difficulties related to vaccine distribution, medicine shortages, cold chain failures and limited supply-chain visibility. These challenges may have contributed to increased interest in digital solutions for monitoring, coordination and operational resilience across healthcare logistics systems. Over time, the literature appears to be moving from narrow discussions focused mainly on blockchain-based traceability toward broader research attention to digital healthcare ecosystems that include artificial intelligence, IoT technologies, predictive analytics and cybersecurity systems. This shift should be interpreted as a thematic pattern in the reviewed literature rather than direct evidence of mature real-world ecosystem implementation. The findings also highlight the multidisciplinary character of this field. This interdisciplinary interpretation is based on journal distribution, keyword clusters, three-fields analysis and author affiliation patterns within the analyzed corpus. Research contributions come from healthcare management, logistics, pharmaceutical sciences, engineering, operations research and information systems, reflecting the growing complexity of digital transformation in healthcare supply chains (52, 53).

### Technological transformation of healthcare and pharmaceutical supply chains

Blockchain-related studies showed high visibility in citation, keyword and co-occurrence analyses, especially in relation to pharmaceutical traceability, counterfeit prevention and secure medicine distribution (54–57). Strong links between blockchain, transparency and cybersecurity within the co-occurrence network suggest that blockchain technologies are frequently discussed as tools for pharmaceutical supply-chain visibility and trust. This visibility should be interpreted as a bibliometric pattern rather than evidence of practical superiority or implementation effectiveness (58–61).

At the same time, Internet of Things technologies are discussed in the reviewed literature as tools for sensor-based monitoring of storage and transportation conditions, particularly for temperature-sensitive medicines and vaccines. IoT-enabled sensors and connected monitoring systems may support cold chain visibility and faster response to disruptions, but their practical effectiveness depends on infrastructure readiness, data integration and implementation capacity.

Another important trend is the growing visibility of artificial intelligence and predictive analytics in healthcare logistics systems. Studies on data analytics, artificial intelligence and machine learning discuss forecasting, inventory optimization and decision-support tools designed to improve operational responsiveness and supply-chain resilience (14–16). However, this bibliometric review identifies AI as a growing research theme and does not directly evaluate its operational effectiveness.

**Figure 10:**
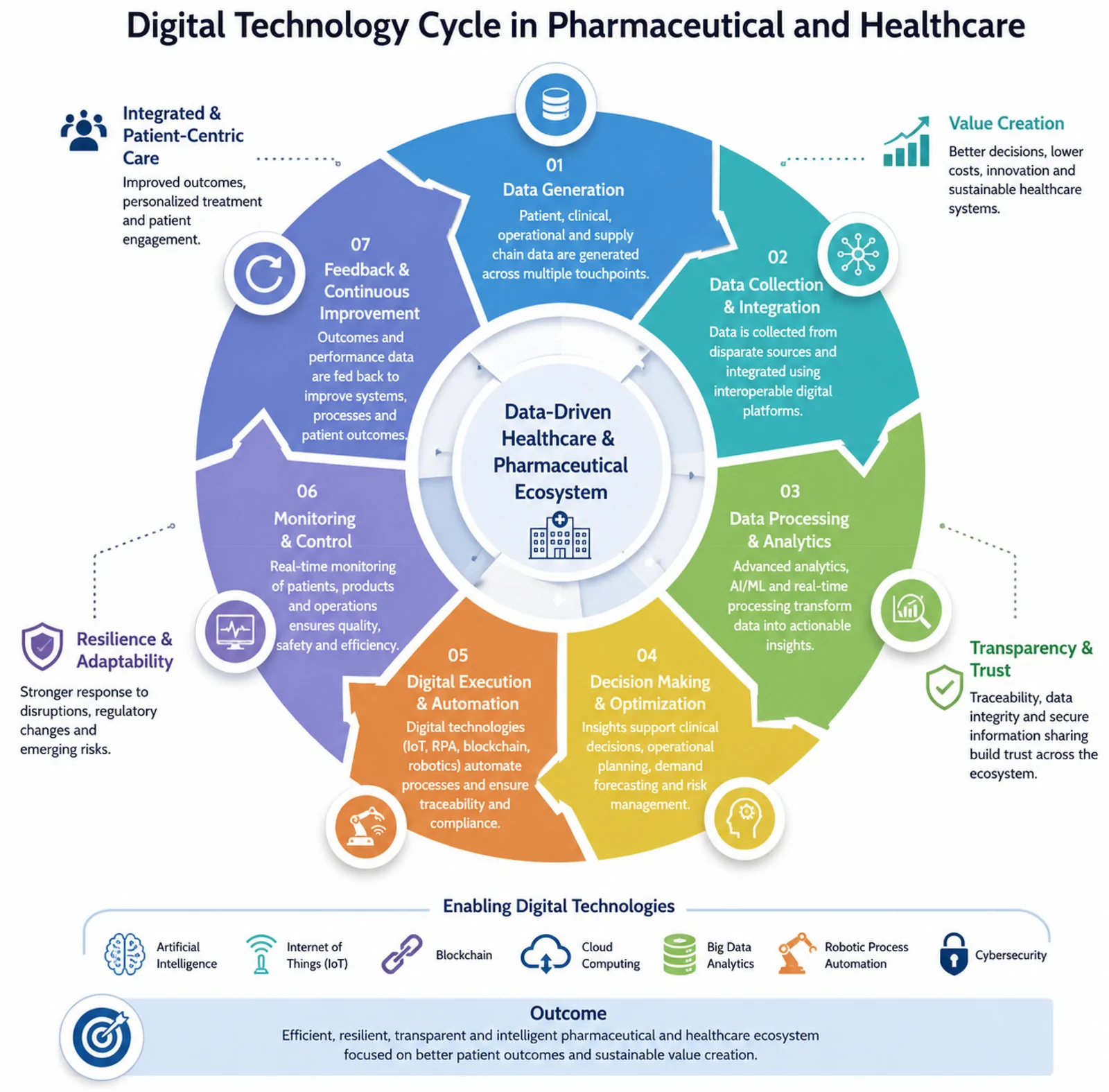
Conceptual cycle of digital technologies in healthcare and pharmaceutical supply chains.

Based on the thematic synthesis of the reviewed literature, a conceptual digital technology cycle was developed, presented in Figure 10, illustrating the interaction between emerging technologies, healthcare logistics processes, pharmaceutical traceability systems, and operational decision-making mechanisms within digital healthcare ecosystems.

The conceptual framework summarizes how digital transformation is discussed in the reviewed literature as moving toward interconnected and data-driven healthcare and pharmaceutical supply chain ecosystems. Blockchain technologies are associated with traceability and transparency, IoT infrastructures with real-time monitoring and cold chain visibility, and artificial intelligence and analytics platforms with forecasting, optimization and decision-support functions. Together, these literature-based associations suggest possible pathways toward more resilient, transparent and adaptive healthcare logistics systems.

### Pharmaceutical traceability, security and regulatory implications

Pharmaceutical traceability and counterfeit prevention emerged as one of the strongest themes within the analyzed literature. The co-occurrence network revealed close relationships between blockchain, traceability, counterfeit drugs, transparency and cybersecurity, reflecting growing research attention to medicine authenticity verification and secure pharmaceutical distribution systems (62). These findings are consistent with broader digital health, Healthcare 4.0 and pharmaceutical supply chain studies that discuss digital maturity, supply-chain visibility, technology adoption and security-oriented transformation in healthcare and pharmaceutical contexts (63–66). The appearance of DSCSA within the thematic network was interpreted as one example of regulatory traceability terminology identified in the keyword structure, rather than as a globally representative or dominant regulatory model. Related studies on resilience, digitalization and Industry 4.0 further support the interpretation that regulatory, technological and organizational factors jointly shape digital transformation in pharmaceutical supply chains (67–69). The reviewed studies suggest that digital traceability systems may support transparency, accountability and supply-chain visibility. However, this bibliometric review does not directly validate operational outcomes. Successful implementation still depends on interoperability standards, regulatory harmonization, secure data-sharing infrastructures and compliance with GDP/GMP/GSP-related requirements.

### Artificial intelligence and intelligent healthcare logistics

Compared with blockchain-centered research, artificial intelligence represents a newer but increasingly visible theme within the analyzed bibliometric corpus. Interest in AI-driven and data-driven healthcare logistics increased after the COVID-19 pandemic, particularly in studies focused on predictive analytics, inventory optimization, disruption management and digital supply-chain resilience (70–72). Many recent publications explore how machine learning, intelligent analytics and technology adoption frameworks may support demand forecasting, supply planning and operational decision-making in healthcare systems. AI-based approaches are also increasingly linked with healthcare resilience, uncertainty management, medicine shortages and changing logistics conditions (73–75). The technology–application analysis suggests that artificial intelligence may play a larger role in future healthcare supply chain research, especially in automation, predictive monitoring and intelligent operational coordination, while related blockchain and traceability studies continue to inform future research on secure pharmaceutical distribution (77, 78). This should be interpreted as a future research direction rather than a confirmed prediction of technological dominance.

### Practical and managerial implications

The findings of this study have important implications for healthcare organizations, pharmaceutical manufacturers, logistics providers and regulatory authorities. The reviewed literature suggests that blockchain technologies may support pharmaceutical traceability, anti-counterfeit protection and secure medicine distribution, while connected identification and monitoring technologies, including RFID- and IoT-based systems, are frequently associated with cold chain visibility, product tracking and connected logistics infrastructures (79–81). Artificial intelligence and big data analytics are increasingly discussed as tools for forecasting accuracy, inventory management and operational decision-making within healthcare logistics systems. However, the practical effectiveness of these technologies depends on data quality, interoperable infrastructures, cybersecurity safeguards, regulatory alignment, workforce readiness and long-term institutional support. Additional recent literature also highlights resilience and agility, security and privacy, greener healthcare supply-chain digitalization, and digitalized healthcare supply-chain design as important implementation priorities (82–85).

### Future research directions

The reviewed literature highlights several promising directions for future research. First, additional studies are needed on integrated digital ecosystems combining blockchain, artificial intelligence, IoT technologies and predictive analytics within unified healthcare logistics infrastructures. Second, more implementation-oriented research is needed to evaluate the real-world effectiveness, scalability and interoperability of digital healthcare supply-chain systems. Current literature remains dominated by conceptual and technology-focused studies, while evidence from practical implementation remains relatively limited.

Future research should also expand the scope of this topic and provide more practical evidence. Future reviews should include more databases and, where possible, non-English publications. Larger and regularly updated datasets would make bibliometric maps and thematic clusters more reliable. More empirical and long-term studies are also needed to evaluate whether blockchain, AI, IoT and analytics-based systems improve real supply-chain outcomes such as traceability, cold chain monitoring, inventory accuracy, response time, cost efficiency and resilience. Finally, comparative studies across different healthcare systems would help explain how infrastructure, regulation and workforce readiness affect digital transformation. Future studies should also examine blockchain-based approaches to reducing pharmaceutical logistics losses and the broader challenges and opportunities of digitalization in healthcare supply chains (86, 87).

Table 7 provides a structured future research agenda based on the bibliometric findings, implementation barriers and methodological limitations identified in this review.

**Table 7:** Future research agenda for digital transformation in healthcare and pharmaceutical supply chains.

Research gap	Future research direction	Expected contribution
Limited database and language coverage	Include additional biomedical, engineering, computer science, regional and non-English sources	Broader and less biased mapping of the research landscape
Small bibliometric corpus	Conduct updated bibliometric reviews with larger datasets and longer observation periods	More stable keyword, citation and collaboration networks
Limited implementation evidence	Conduct empirical case studies, field evaluations and longitudinal studies	Stronger evidence on real-world effectiveness and scalability
Technology integration remains underexplored	Study integrated blockchain-AI-IoT-analytics ecosystems	Better understanding of interoperable digital supply-chain architectures
Regulatory and cybersecurity challenges	Examine governance, privacy, compliance and cybersecurity frameworks	Practical guidance for safe and compliant digital implementation
Limited evidence from developing countries	Compare implementation barriers across different health-system contexts	More context-sensitive digital transformation strategies

### Limitations

This study has several limitations that should be considered when interpreting the findings. First, the analysis was limited to publications indexed in Scopus, Web of Science and PubMed. Although these databases represent major sources of scientific literature, relevant publications indexed in engineering-focused, computer science, regional or grey-literature databases may not have been included. This may affect the completeness of the bibliometric map.

Second, only English-language peer-reviewed journal articles were analyzed. This restriction improved consistency across bibliometric indicators but may have introduced language bias and reduced the representation of region-specific research published in other languages.

Third, the final bibliometric corpus consisted of 83 journal articles. Although this corpus was suitable for exploratory bibliometric mapping, its relatively small size may limit the stability of some collaboration networks, less frequent keyword clusters and technology–application associations.

Fourth, conference proceedings, book chapters and non-peer-reviewed documents were excluded. This improves methodological consistency but may underrepresent fast-moving technology-related research, especially in areas such as artificial intelligence, blockchain, IoT and digital logistics.

Fifth, keyword standardization was performed manually to harmonize terms such as AI/artificial intelligence and IoT/Internet of Things. Although this improved interpretability, some terminology variation may remain. Finally, because this study was designed as a bibliometric mapping review, it did not evaluate the effectiveness of clinical interventions, operational outcomes or real-world implementation performance. Therefore, traditional clinical risk-of-bias tools were not applicable, and the findings should be interpreted as patterns of scientific production, citation visibility and thematic association rather than direct evidence of practical effectiveness.

## Conclusion

This study provides a bibliometric overview of research on digital transformation and emerging technologies in healthcare and pharmaceutical supply chains. Within the analyzed corpus, scientific activity increased after 2020, suggesting growing research attention to resilient, transparent and digitally connected healthcare logistics systems.

Blockchain showed strong visibility within the analyzed literature, particularly in relation to pharmaceutical traceability, transparency and counterfeit prevention. At the same time, artificial intelligence, IoT systems and predictive analytics emerged as important research themes associated with healthcare monitoring, forecasting and operational decision-making.

Thematic evolution suggests growing research attention to integrated digital healthcare supply chain models combining intelligent analytics, connected monitoring systems and resilient supply-chain infrastructures. However, these findings should be interpreted as bibliometric and thematic patterns within the analyzed corpus rather than direct evidence of real-world technology effectiveness.

Despite growing research activity, the literature continues to highlight several important implementation challenges, including interoperability limitations, cybersecurity concerns, regulatory complexity and shortages of digital expertise. Future research should therefore move beyond conceptual and bibliometric mapping toward implementation-oriented, comparative and outcome-based studies.

## Data Availability

The original contributions presented in the study are included in the article/Supplementary Material, further inquiries can be directed to the corresponding author.

## Supplementary material

Supplementary Table S1 Full search strategy across Scopus, Web of Science and PubMed.

Supplementary Dataset S2 Final bibliometric corpus of 83 included records used for bibliometric mapping, annual scientific production, source analysis, citation analysis, keyword analysis, temporal thematic evolution and technology–application interpretation.
