# Supplementary material for: Digital transformation and emerging technologies in healthcare and pharmaceutical supply chains: a bibliometric mapping review
Source: Front Digit Health. 2026 Aug 27;8:1884773. doi: 10.3389/fdgth.2026.1884773 (PMC13558156; doi:10.3389/fdgth.2026.1884773)
Supplement: Supplementary file 1 [file Table1.docx]

Supplementary Material

# 1 Supplementary Figures and Tables

**Supplementary Table S1.** Full search strategy across databases.

| **Database** | **Search Query** |
| --- | --- |
| **Scopus** | TITLE-ABS-KEY( ( "healthcare supply chain" OR "health care supply chain" OR "medical supply chain" OR "pharmaceutical supply chain" OR "biopharmaceutical supply chain" OR "healthcare logistics" OR "pharmaceutical logistics" OR "drug distribution" OR "vaccine supply chain" ) AND ( "digital transformation" OR digitalization OR digitization OR "digital technolog*" OR "smart supply chain" OR "Industry 4.0" OR "Industry 5.0" ) AND ( "artificial intelligence" OR AI OR "machine learning" OR "deep learning" OR "internet of things" OR IoT OR blockchain OR "big data" OR "data analytics" OR "predictive analytics" OR "digital twin*" OR "cloud computing" OR automation OR sensor* OR RFID ) ) |
| **Web of Science** | TS=(  (  "healthcare supply chain" OR "health care supply chain" OR "medical supply chain" OR "pharmaceutical supply chain" OR "biopharmaceutical supply chain" OR "healthcare logistics" OR "pharmaceutical logistics" OR "drug distribution" OR "vaccine supply chain"  )  AND  (  "digital transformation" OR digitalization OR digitization OR "digital technolog*" OR "smart supply chain" OR "Industry 4.0" OR "Industry 5.0"  )  AND  (  "artificial intelligence" OR AI OR "machine learning" OR "deep learning" OR  "internet of things" OR IoT OR blockchain OR "big data" OR "data analytics" OR "predictive analytics" OR "digital twin*" OR "cloud computing" OR automation OR sensor* OR RFID  )  ) |
| **PubMed** | ( ("Supply chain"[Title/Abstract] OR "pharmaceutical supply chain"[Title/Abstract] OR "healthcare supply chain"[Title/Abstract] OR "drug distribution"[Title/Abstract]) ) AND ( ("Digital Technology"[MeSH] OR "Artificial Intelligence"[MeSH] OR "Machine Learning"[MeSH] OR "Internet of Things"[Title/Abstract] OR blockchain[Title/Abstract] OR "big data"[Title/Abstract]) OR ("digital transformation"[Title/Abstract] OR digitalization[Title/Abstract] OR digitization[Title/Abstract] OR "digital technolog*"[Title/Abstract] OR "Industry 4.0"[Title/Abstract]) ) AND ( ("Health Services"[MeSH] OR "Pharmaceutical Services"[MeSH]) OR (healthcare[Title/Abstract] OR "health care"[Title/Abstract] OR medical[Title/Abstract] OR pharmaceutical[Title/Abstract] OR biopharmaceutical[Title/Abstract]) ) |
